# Past accomplishments and future challenges of the multi-omics characterization of leaf growth

**DOI:** 10.1093/plphys/kiac136

**Published:** 2022-03-24

**Authors:** Aleksandra Skirycz, Alisdair R Fernie

**Affiliations:** 1 Max-Planck-Institute of Molecular Plant Physiology, Potsdam-Golm 14476, Germany; 2 Boyce Thompson Institute, Ithaca, New York 14853, USA; 3 Cornell University, Ithaca, New York 14853, USA

## Abstract

The advent of omics technologies has revolutionized biology and advanced our understanding of all biological processes, including major developmental transitions in plants and animals. Here, we review the vast knowledge accumulated concerning leaf growth in terms of transcriptional regulation before turning our attention to the historically less well-characterized alterations at the protein and metabolite level. We will then discuss how the advent of biochemical methods coupled with metabolomics and proteomics can provide insight into the protein–protein and protein–metabolite interactome of the growing leaves. We finally highlight the substantial challenges in detection, spatial resolution, integration, and functional validation of the omics results, focusing on metabolomics as a prerequisite for a comprehensive understanding of small-molecule regulation of plant growth.

## Teaser

The advent of omics technologies advanced our understanding of molecular mechanisms underlying leaf growth.

## Fundamentals of leaf growth

Herein, we first introduce basic facts about leaf growth, focusing on the practical implications for omics characterization. We will concentrate on Arabidopsis thaliana (“Arabidopsis”) and *Zea mays* (“maize”) as established models for dicot and monocot leaf growth. However, it has to be noted that tip-to-base differentiation gradient, common to the model plant species and outlined below, is not necessarily universal among eudicots (Das Gupta and Nath, 2015).

The growth of plant leaves is governed by cell division followed by cell expansion (Donnelly et al., 1999; Sylvester and Smith, 2009). Leaves initiate at the flank of the shoot apical meristem. Initially, the growth of a new leaf is driven by rapid cell proliferation at the division zone. At a specific time after primordium initiation, a so-called cell cycle arrest front (AF) is established at a fixed position in the leaf primordium (Kazama et al., 2010; Andriankaja et al., 2012). Cells exit mitosis and start expanding by means of vacuolar growth, also referred to as differentiation, with the exception of meristemoid cells that retain the ability to proliferate (Donnelly et al., 1999). In many plant species including Arabidopsis (Barow and Meister, 2003), exit from the mitotic cell cycle is accompanied by the onset of endoreduplication and enlargement of nuclei (Galbraith et al., 1991; John and Qi, 2008; Tsukaya, 2019). It was postulated that proliferation is driven by a mobile growth factor (MGF) coming from the leaf base (Kazama et al., 2010). As the leaf grows, cells at the tip move away from the MGF and the AF is formed. AF is further maintained by the balance of the antagonistic regulators acting at the proliferation-expansion transition zone (Kalve et al., 2014). Differentiated cells in turn become the source of a putative mitotic arrest signal that is generated at the leaf-tip (Nath et al., 2003). In Arabidopsis and maize, meristematic region at the leaf base maintains a constant size and proliferation ability followed by abrupt arrests throughout the basal region (Sylvester and Smith, 2009; Andriankaja et al., 2012). However, whereas in Arabidopsis, the meristem arrest occurs shortly after the establishment of the AF, in maize the leaf meristem persists for much longer, and meristem arrest is linked with the cessation of leaf growth (Fournier et al., 2005). Analogously, cells cease to expand and reach maturity in a tip-to-base fashion in both Arabidopsis and maize, the transition being much more rapid in Arabidopsis. Because of this difference in the timing of meristem disappearance, leaf growth in Arabidopsis is described as temporally-, whereas in maize as spatially- regulated (Nelissen et al., 2016), which has major implication for profiling studies. In order to capture the molecular events associated with transitions from proliferation into expansion and maturity, in Arabidopsis complete leaves are typically harvested at multiple days following primordium initiation. For instance, in Arabidopsis, the third true leaf harvested from 8-d-old plants is fully proliferating, at Day 14 expanding and at Day 21, mature nongrowing (Skirycz et al., 2010a, 2010b). Alternatively, the different number leaves, corresponding to the different growth stages are harvested at a single time-point (Efroni et al., 2008). Compared to Arabidopsis, in maize, a growing leaf is cut into horizontal stripes that correspond to the different growth stages, and transition zones (Avramova et al., 2015; Czedik-Eysenberg et al., 2016). The big advantage of working with maize over Arabidopsis leaves is the large amount of material, which can be harvested from a relatively few plants (Sprangers et al., 2016), related to the difference in leaf size. Fully proliferating Arabidopsis leaves are less than 0.1 mm^2^ in size and have to be dissected under binocular. Whereas the use of the RNA later has in the past facilitated transcriptomics analysis (Efroni et al., 2008) of the Arabidopsis leaf primordia including a time-resolved response to the osmotic (Skirycz et al., 2011a, 2011b) and drought stresses (Clauw et al., 2016), proteomics and metabolomics analysis are affected by the time it requires to harvest sufficient amount of material.

Which mechanisms determine the final leaf size constitutes an open question in plant biology. From a cellular perspective, final leaf size results from a combination of cell number and cell size. Cell number may be dictated by the initial primordium size, proliferation rates (doubling time), the timing of the transition between proliferation and expansion, and meristemoid activity. Analogously cell size is related to the rate and duration of expansion (Donnelly et al., 1999; Sylvester and Smith, 2009; Gonzalez et al., 2012; González and Inzé, 2015). A recent meta-analysis of the growth and cellular phenotypes of the leaf-size mutants, natural accessions, and environmental conditions point to the meristem size, timing of the exit from proliferation and duration of leaf expansion as the prime determinants of the final leaf size in both monocots and dicots (Gázquez and Beemster, 2017). For example, delayed meristem arrest results in bigger leaves, and was reported for loss and gain of function Arabidopsis mutants of known leaf growth regulators such as *AINTEGUMENTA* (Mizukami and Fischer, 2000), ubiquitin Receptor DA1 (Vanhaeren et al., 2016), and *miR319a* (Efroni et al., 2008)*.* Environmental stress has in turn been linked to the reduction of leaf size by negatively affecting both cell number and size (Lecoeur et al., 1995; Tardieu et al., 2000, 2011; Aguirrezabal et al., 2006; Koch et al., 2019). Stress can induce meristem arrest (Skirycz et al., 2011a, 2011b; Nelissen et al., 2018) but can also increase the duration of cell divisions (Koch et al., 2019). Related and critical aspect of leaf growth is its plasticity which buffers for the environmental and genetic perturbation that interfere with proliferation or expansion to ensure an optimal leaf size (Hisanaga et al., 2015). For instance, Arabidopsis mutants characterized by the decreased cell numbers are characterized by larger cells (Ferjani et al., 2007, 2013). Remarkably, the exact mechanism by which the compensation is achieved differs for the various mutants, ranging from the larger size at cell division to an extended duration of growth (Ferjani et al., 2007, 2013). Analogously, inhibition of leaf growth in response to stress can be compensated for by an extended duration of growth (Aguirrezabal et al., 2006; Nelissen et al., 2018) or increased meristemoid activity (Skirycz et al., 2011a, 2011b).

**Advances**

- Leaf growth has been studied extensively at the level of transcriptional regulation, particularly in the model C3 plant Arabidopsis and model C4 plant maize.
- Recently, these studies have been complemented by surveys of the dynamic shifts at the level of the proteome and metabolome.
- When evaluated alongside one another these studies provide a detailed overview as to how leaf growth is programmed, although the underlying mechanisms are, as yet, only partially solved.

Because, and as demonstrated above, final leaf size is determined by the combination of the different and highly dynamic cellular processes, molecular characterization of leaf growth is usually accompanied by detailed growth analysis to ensure correct interpretation of the omics data. Historically, the growth status of a leaf or a leaf region has been assessed based on the microscopic observation of the epidermal cells. The transition between cell division and cell expansion can be visualized using the mitotic cyclin marker lines, and by assessing cell shape (Donnelly et al., 1999). In dicots, expanding cells are known for their characteristic highly lobed, jigsaw-like appearance, whereas in monocots, the cells become elongated. Furthermore, the size of the basal meristem reason can be estimated using DAPI staining (Sprangers et al., 2016). The time-resolved kinematic analysis of leaf growth and the underlaying changes in cell number and cell size, however, highly laborious, remains the method of choice (Fiorani and Beemster, 2006). Cell ploidy levels are typically measured using flow-cytometry and onset of endoreduplication, in plants such as Arabidopsis, serves as a good proxy for differentiation.

## Transcriptomics and proteomics of leaf growth

**Figure 1:**
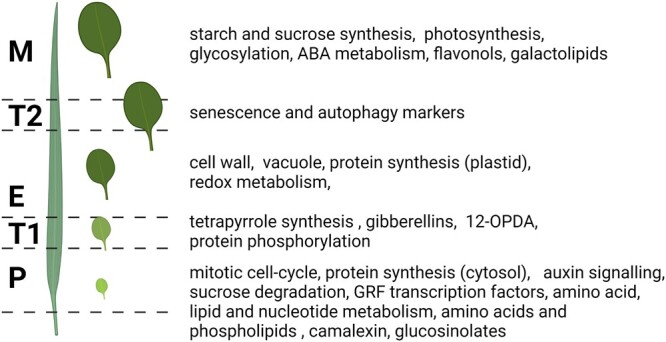
Leaf growth is associated with changes at the transcriptome, proteome, and metabolome levels. Arabidopsis and maize are established models for dicot and monocot leaf growth, characterized by tip-to-base differentiation gradients. Leaves or leaf zones corresponding to the different growth stages and transition zones are used as a starting material for molecular characterization. Transcriptomics, proteomics, and metabolomics analysis of leaf growth in maize and Arabidopsis revealed major transcriptional make-over reflected at the protein and metabolite levels. P, proliferation; T1, transition from proliferation to expansion; E, expansion; T2, transition from expansion to maturity; M, maturity. Created using Biorender.com.

To date, there are numerous published examples where single-omics have been used to characterize the regulation of leaf growth in both Arabidopsis and maize. In the next two chapters, we will use selected examples to illustrate what was learned by combining transcriptomics, proteomics, and metabolomics analysis of the different leaf growth stages and transition zones (summarized in Figure 1). However, we will focus mainly on the studies looking at wild-type plants grown under optimal conditions; we will refer to experiments incorporating stress treatments, leaf growth mutants, and natural accessions.

Transcriptomics was first among the different omics studies used to look for molecular signatures of the different growth stages. For instance, Beemster et al. (2005) combined growth, cellular, and ploidy analysis with the 6k-cDNA microarray to characterize the transcriptome of the Arabidopsis leaves harvested every second day from proliferation into maturity. Obtained results identified major changes, with more than 30% of measured transcripts displaying differential accumulation across leaf growth. Transcript data discriminated between the proliferating, expanding, and mature leaves, and were used to delineate a set of proliferation-specific transcripts, including multiple genes encoding for the core cell cycle proteins. The predominant gene expression pattern characterized by increase or decrease of hundreds of transcripts from proliferation into maturity has also been repetitively reported by the later studies (Li et al., 2010a, 2010b; Efroni et al., 2008; Andriankaja et al., 2012; Baerenfaller et al., 2012; Nelissen et al., 2018; Mergner et al., 2020). For instance, in the maize leaf, the gradient of gene expression across the entire leaf but also within the division and elongation zones has been elegantly demonstrated by Nelissen et al. (2018). As a major generalization, the transition from proliferation into expansion is accompanied by the decreased expression of genes important for the mitotic divisions and cytoplasmic growth such as B-cyclins, components of the mitotic spindle and ribosomal subunits, but increased expression of genes associated with the expansion and establishment of the photosynthesis, such as aquaporins, expansins, and enzymes of the Calvin–Benson–Bassham cycle.

In fact, the transition from proliferation to expansion occurs simultaneously with the establishment of photosynthesis, chloroplast biogenesis, and maturation starting at the leaf-tip (Andriankaja et al., 2012). Intrigued by the expression of tetrapyrrole biosynthesis genes which proceeded accumulation of other photosynthetic transcripts Andriankaja et al. (2012) monitored leaf differentiation onset in plants subjected to the norflurazon treatment. Inhibition of the plastidial development was associated with a longer proliferation implicating as of yet unknown plastid derived signals in the regulation of leaf growth (Andriankaja et al., 2012). Analogously to Arabidopsis, expression of the genes encoding for enzymes responsible for the production of precursors of photosynthetic pigments was also reported in the maize distal meristem zone (Nelissen et al., 2018).

Whereas a plastidial derived signal has been proposed to play a role in meristem arrest, senescence- and autophagy-associated transcripts were found to accumulate in the expanding leaves prior to transition to maturity (Baerenfaller et al., 2012; Nelissen et al., 2018). However, while this potentially reflects an enhanced need for respiratory substrates in order to complete the process of leaf maturation, it may also point to the involvement of autophagy-derived signals in the cessation of leaf growth. It has to be noted that counterintuitively, given its role for the final leaf size, the regulation of transition from expansion to maturity had received relatively little interest in the past.

The transcriptional make-over associated with growth progression is well aligned with the genetic evidence. Many of the known leaf growth regulators are in fact transcriptional regulators (Sarvepalli et al., 2019). In Arabidopsis, the prominent place belongs to the family of GROWTH REGULATING FACTORS (GRFs) and CINCINNATA-like TEOSINTE BRANCHED 1, CYCLOIDEA, PCF1 (TCPs) transcription factors (Vercruyssen et al., 2015). GRFs expression is associated with the meristematic cells and is repressed by the microRNA396, a negative regulator of leaf growth (Liu et al., 2009). Importantly GRFs work in a complex with other transcriptional regulators including co-activator ANGUSTIFOLIA3 (AN3) (Horiguchi et al., 2005) and several components of the SWItch/Sucrose Non-Fermentable (SWI/SNF) chromatin remodeling complex (Vercruyssen et al., 2014). In comparison, TCP TFs are growth repressing factors associated with the onset of differentiation, partly via negative regulation of GRF expression (Sarvepalli and Nath, 2018). As would be expected, GRFs, ANT3, and TCP are among genes displaying differential expression across at the transition from proliferation to expansion (Andriankaja et al., 2012). Targets of the key TFs associated with leaf growth, such as AN3 (Vercruyssen et al., 2014), were characterized by tandem chromatin affinity purification (AP) and chromatin immunoprecipitation (ChIP-seq) studies aimed at the genome-wide characterization of the TFs binding sites. By far the most extensive study to date used ChIP-seq to identify binding site of 104 TFs to reconstruct transcriptional regulatory network in maize leaves (Tu et al., 2020). The resulting network covered over 77% of the leaf genes, with single genes and pathways regulated by dozens of TFs in contrast to a few master regulators. Finally, additional layer of transcriptional regulations, associated with DNA methylation was reported in maize leaves by profiling four different growth zones. The level of methylation corresponded to the onset of cellular differentiation, supporting the role of epigenetic modification in controlling plant growth (Candaele et al., 2014).

Transcriptional regulation has been also implicated in the leaf growth arrest in response to stress. Time-resolved transcriptomic analysis of plants subjected to the osmotic stress, for instance, revealed a highly interconnected and dynamic regulatory network composed of multiple transcription factors, which is specific to the growing leaves (Skirycz et al., 2011a, 2011b). A central role in this network belongs to the ETHYLENE RESPONSIVE FACTORS (ERFs), which were shown to control growth via regulation of gibberellin metabolism, and consequently DELLA stability and signaling (Dubois et al., 2013, 2015; Van den Broeck et al., 2017). Whereas overexpression of the selected ERF TFs, such as for example, *ERF6*, leads to growth inhibition, loss-of-function mutants are characterized by a reduced growth penalty in response to stress (Dubois et al., 2013). In a similar vein (Baerenfaller et al., 2012) analyzed, the transcriptome of Arabidopsis leaves at four different growth stages harvested from plants grown under optimal and moderate drought conditions. Obtained results revealed clear difference between osmotic and drought treatments and were corroborated further by the transcriptomic analysis of growing leaves harvested from the multiple Arabidopsis accession subjected to mild-drought (Clauw et al., 2016). Nevertheless, and strikingly, both mild-osmotic and drought stresses (Skirycz et al., 2011a, 2011b; Baerenfaller et al., 2012) were associated with a molecular signature associated with carbon abundance, rather than carbon depletion. This attested to stress-associated leaf growth repression being an important adaptation mechanism rather than simply a result of carbon limitation.

Transcriptomic analysis of the growing leaves was followed-up by proteomics characterization in Arabidopsis and in maize (Majeran et al., 2010; Baerenfaller et al., 2012; Mergner et al., 2020; Omidbakhshfard et al., 2021). It could be demonstrated that with few prominent exceptions such as subunits of the ATP synthase complex (Baerenfaller et al., 2012; Omidbakhshfard et al., 2021) transcript and proteins levels are well associated. Such good association points to protein translation being the primary determinant of protein abundance and composition during plant growth. As in case of transcripts, most of the differential proteins are characterized by a gradual increase or decrease across leaf growth stages. Whereas proteins associated with cell division and cytoplasmic growth, including cellulase synthase complex and cytosolic ribosomal subunits, are highly abundant in the leaf meristem, proteins associated with expansion, such as vacuolar ATPases, accumulate in the expanding cells (Mergner et al., 2020; Omidbakhshfard et al., 2021). Moreover, while meristematic zone is characterized by a sink-like metabolism, mature nongrowing cells acquire full photosynthetic competency and become a carbon source (Majeran et al., 2010). Proteins associated with plastid development, light, and dark reactions of photosynthesis, and starch metabolism start to accumulate around the proliferation to expansion transition, and many reach their maximum abundance in the mature leaf (Majeran et al., 2010).

Furthermore, comparative analysis of the proteomics and phosphoproteomics datasets generated for maize leaves reveled prevalent role of phosphorylation in the growing versus mature cells (Facette et al., 2013). Intriguingly, and in contrast to protein abundance, phosphorylation events were especially common during transition from proliferation to expansion, suggesting that the differentiation is regulated by protein phosphorylation. In accordance, differentially phosphorylated proteins were enriched in proteins associated with signaling and regulation.

## Metabolomics of leaf growth

Since its advent over 20 years ago metabolomics has been extensively employed as a tool both to gain insight into the metabolic response to environmental or genetic variance (Sumner et al., 2003; Saito and Matsuda, 2010a, 2010b; Alseekh and Fernie, 2018; Zhu et al., 2018; Fang et al., 2019; Kosmacz et al., 2020). It has, furthermore, been much utilized to further our understanding of the metabolic shifts that characterize the development of fruits (Carrari et al., 2006; Klie et al., 2014; Roch et al., 2020) and seeds (Fait et al., 2006; Angelovici et al., 2009, 2010) and to a lesser extent other organs including roots (Moussaieff et al., 2013) and flowers (Borghi et al., 2017; Borghi and Fernie, 2017). Somewhat surprisingly the application of metabolomics to understand leaf growth is considerably less common with the majority of comprehensive studies in this vein being focused on the final developmental stage—senescence (Watanabe et al., 2013). While considerable insight into this process has been achieved this is the subject of several detailed reviews (Lim et al., 2003, 2007; Schippers, 2015; Woo et al., 2019) and outside the scope of this article in which we intend to cover the timespan between leaf initiation and maturity. Despite the relative paucity of studies in this respect, there are a handful of insightful papers which warrant discussion (Skirycz et al., 2010a, 2010b; Pick et al., 2011; Wang et al., 2014; Kanojia et al., 2020; Omidbakhshfard et al., 2021)), whilst further useful observations could be found in several other less detailed studies (Fukushima and Kusano, 2014; Schulz et al., 2014; Allwood et al., 2015). Given that they are distinctive we will initially discuss the metabolic shifts underlying the development of leaves in C3 and C4 species separately and then compare and contrast them.

The metabolic changes that occur during C3 metabolism had until recently received relatively little attention. Arguably, the best characterization until this decade was provided by a pair of studies concerning the response of Arabidopsis to mild osmotic stress (Skirycz et al., 2010a, 2010b, 2011a, 2011b). These studies whilst not focused entirely on leaf growth nevertheless revealed considerable insight into this process demonstrating that metabolic response of growing versus mature leaves are very different. For instance, proline, arginine, ornithine, and putrescine, metabolites historically associated with plant response to salt stress, accumulated in the mature but not in the growing leaves. In a similar vein the recent study of Kanojia et al. (2020) revealed following the analysis of progressively aging but not yet senescing Arabidopsis rosette leaves that a decrease in primary metabolites that provide protection against oxidative stress (Obata and Fernie, 2012) is consistent with the concomitantly observed transcriptional stress signature. The transcriptional stress signature covers a set of genes that have previously been identified across multiple datasets to be highly responsive under conditions of stress (Gadjev et al., 2006). The fact that signatures of stress are observed at both the metabolome and transcriptome level strongly suggests that its onset considerably proceeds that of senescence. The far more comprehensive study of Omidbakhshfard et al. (2021) revealed major changes in metabolic composition across early Arabidopsis growth. Of the 285 annotated small-molecular compounds covering primary and specialized metabolites and lipids measured across six consecutive days of leaf growth, 145 were characterized by a differential accumulation. With few prominent exceptions differential compounds displayed either proliferation- or expansion-specific accumulation patterns. Proliferation-specific compounds included amino acids, nucleotides, phospholipids, and intriguingly also glucosinolates and camalexin, which are well known specialized defense compounds. In comparison, expansion-specific metabolites comprised amino acid proline, TCA cycle intermediates, and thylakoid membrane lipids. One of the notable observations that came from the metabolomic analysis of the Arabidopsis leaves was a short-term accumulation of 12-oxophytodienoic acid (12-OPDA), specifically at Day 11, which coincides with the disappearance of the leaf meristem (Omidbakhshfard et al., 2021). 12-OPDA levels measured at Day 11 were approximately eight times higher than at Days 10 and 12. 12-OPDA is the precursor of a plant hormone jasmonic acid and also a signaling molecule in its own right (Wasternack and Strnad, 2016; Maynard et al., 2018). A major peak of 12-OPDA accumulation coinciding with the disappearance of the leaf meristem, suggested a role of oxylipins in the leaf meristem arrest.

The above provides the state-of-the-art of our knowledge of C3 leaf growth. That of C4 leaf growth is nicely defined by three papers published within the last ten years which both confirmed and extended the findings of a range of earlier studies. In the first of these Pick et al., characterized the developmental gradient of a maize leaf by systematically evaluating 10 continuous leaf slices from the point of emergence to the base of the leaf in order to study the development of the organ (Pick et al., 2011). Using transcriptome analysis alongside photosynthetic characterization they found that the maize leaf undergoes a source-sink transition without an intermediate phase of C3 or C2 photosynthesis. Moreover, these analyses coupled with metabolome and transcriptome analyses revealed that these displayed continuous gradients also suggesting that the morphogradient was the determining factor of developmental stage. These analyses did however suggest the presence of some transcription factors that were involved in the establishment of C4 photosynthesis, with a considerable number of further candidates being identified in the C4 rice project (Sedelnikova et al., 2018). A follow up study (Czedik-Eysenberg et al., 2016), investigated the response of metabolome, transcriptome, and polysome loading at dusk, dawn, and various time points for an extended night tracking whole leaf elongation over this time-course. They found that starch and sugar levels are exhausted by dawn but only following the extension of night and exclusively in the elongation and division zones. However, the fact that the global metabolome and transcriptome dynamics follow the changes in sucrose and polysome loading also suggests that growth processes are determined by local carbon status. Finally, the study of Wang et al. (2014) compared and contrasted leaf gradients as described above in the Pick study between maize and the C3 plant rice using a statistical method they named the uniform development model allowing the identification of further transcription factors and cis-regulatory elements that might be responsible for differences in photosynthesis as well as carbon and nitrogen metabolism between these species. A further difference between C4 grasses and C3 plants such as Arabidopsis is the extent of endoreduplication which is far lower in many C4 grasses.

In a similar vein, comprehensive hormone profiling of the maize leaves revealed an accumulation of auxin and cytokinin in the leaf meristem (Nelissen et al., 2012). Intriguingly proliferation to expansion transition zone was characterized by the accumulation of gibberellic acid (GA_1_). Maize mutants characterized by low GA_1_ levels or constitutively active DELLA proteins are characterized by shorter meristem and consequently smaller leaves, whereas elevated levels of GA_1_ has the opposite effects yielding bigger plants. Based on the obtained results it was concluded that GA signaling contributes to the cell-cycle exit and onset of cell expansion. The involvement of DELLA proteins in the growth regulation goes beyond optimal conditions, as DELLA proteins have been also implicated in the growth repression in response to stress in both maize and Arabidopsis (Achard et al., 2009; Claeys et al., 2012; Fina et al., 2017; Nelissen et al., 2018).

## The time is now: dissecting protein–protein and protein–metabolite interactomes of the growing leaves

**Figure 2:**
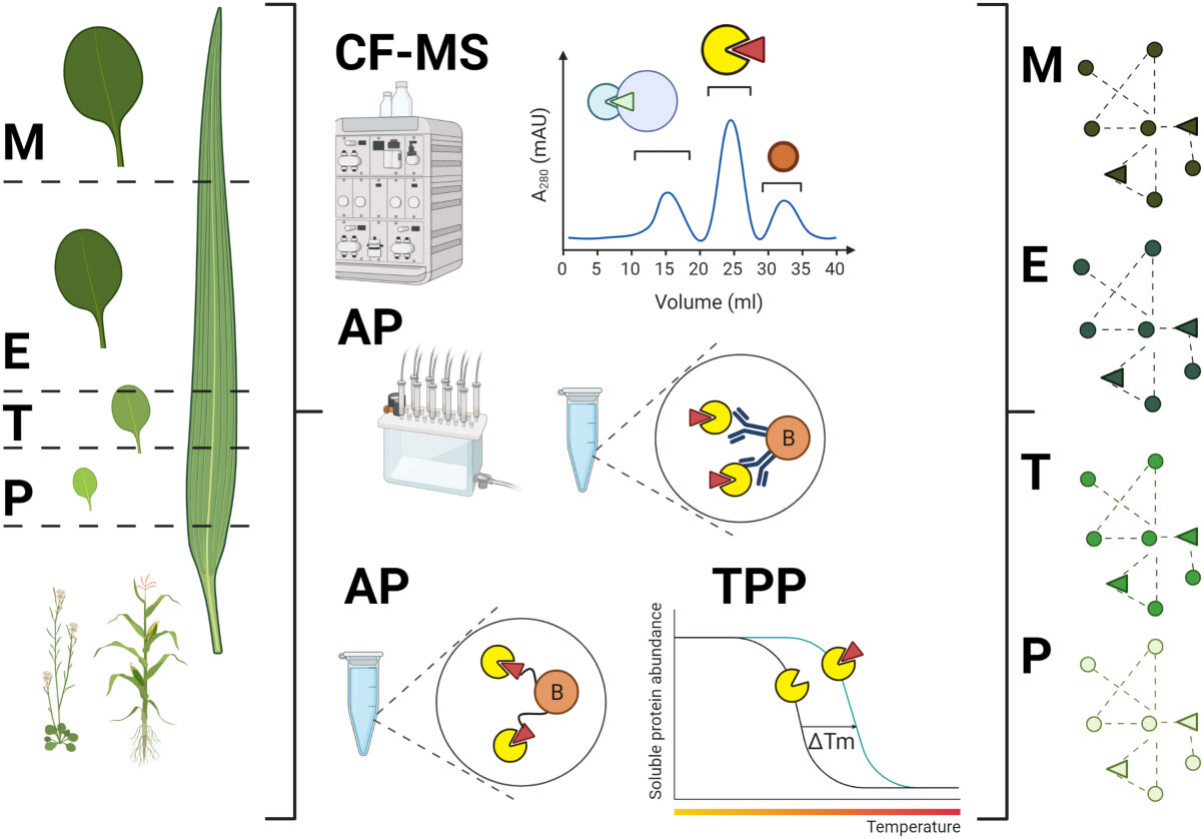
Different strategies to dissect the protein–metabolite interactome of the growing leaves. Left panel, schematic representation of the various growth stages in Arabidopsis and maize leaf. P, proliferation; T, transition; E, expansion; M, maturity. Middle panel, schematic representation of the different experimental approaches. Circles represent proteins; triangles represent metabolites; circle with B stands for bead. AP starting with either protein or protein as a bait. Right panel, schematic representation of the protein–protein–metabolite interactome, which we expect will substantially significantly differ between the growth stages. Created using Biorender.com.

Proteins and metabolites rarely act on their own but as part of the multi-component complexes. As outlined above, leaf growth is associated with profound changes to both proteome and metabolome. Hence, we expect that transition from proliferation to expansion and subsequent cessation of leaf growth will be associated with a substantial re-wiring of the protein–protein and protein–metabolite interactomes. We also anticipate that many of the protein–protein (PPI) and protein–metabolite interactions (PMI) will be regulatory and that many more small molecules than described today will be involved in regulating the different aspects of plant growth. In this chapter, we will give examples that support the above notion. We will also highlight biochemical approaches that rely on mass spectrometry (MS)-based detection of interactors for metabolome- and proteome-wide characterization of PPIs and PMIs (see Figure 2).

In the past, AP coupled to MS detection (AP/MS), where the protein of interest is used as a bait to fish-out interactors, has been successfully used to identify protein partners of proteins associated with plant growth. Most notably, systematic characterization of the protein partners of nearly 100 core cell cycle proteins (Van Leene et al., 2010), specifically in the Arabidopsis cell cultures, revealed a highly interconnected PPI network and uncovered growth regulators, such as SAMBA (Eloy et al., 2012) and bZIP29 TF (Van Leene et al., 2016). Cell cultures are an excellent source of starting material for several reasons. They are easy and fast to transform, provide ample protein and are a perfect model for meristematic cells. However, not all interactions can be retrieved using cell cultures. This is well illustrated by an AP/MS study focused on the maize AN3 protein. ZmAN3 was expressed in the growing leaves and the AP/MS pull-down was performed using either dividing or expanding leaf material. Intriguingly, whereas in the leaf meristem, ZmAN3 was found to interact with positive regulator of cell proliferation ZmGRF1 in the expanding cells, ZmAN3 interacted with ZmGRF10 a negative regulator of divisions (Nelissen et al., 2015). Such competitive binding across a leaf axis was speculated to contribute to the establishment of the AF. In comparison, AN3 AP/MS experiments using Arabidopsis cell cultures captured the SWI/SNF chromatin remodeling complex but not the GRF TFs (Vercruyssen et al., 2014). AP/MS from plant leaf material was also successfully used to identify interactors of ubiquitin receptor DA1 (Vanhaeren et al., 2020). UBR12 and UBR13 are ubiquitin proteases and negative regulators of DA1, which negatively regulates leaf growth. The different technical aspects of the AP/MS, such as choice of the suitable tag and antibody, the use of negative controls, and data analysis, have been extensively reviewed by Bontinck et al. (2018).

AP/MS proved highly successful in identifying protein–protein complexes (see above). However, as every approach starts with cellular lysate and requires a rather lengthy purification procedure, AP/MS may miss transient and low-affinity interactions. A viable alternative to AP/MS, which circumvents such limitations, is proximity biotinylation (Mair et al., 2019; Zhang et al., 2019). Here the protein of interest is fused to a biotin ligase. Proteins found in the proximity of a bait get permanently biotinylated and are then enriched with streptavidin beads and identified by MS. First developed for animal cells (Branon et al., 2018), proximity labeling has by now been adapted to plants (Arora et al., 2020).

Furthermore, although historically used for the PPIs, AP/MS is also suitable to retrieve small-molecule partners of the protein of choice (Luzarowski et al., 2017). The method’s sole iteration involves removing the detergents, which may interfere with the downstream MS-based metabolomics. For instance, a systematic characterization of the 103 yeast kinases identified the small-molecule lipid ligands for the 21 of the studied proteins (Li et al., 2010a, 2010b) attesting to the prevalent however vastly uncharacterized role of small-molecules in regulating protein activities. Related, we speculate that known small-molecule regulators of leaf growth constitute just a tip-of the iceberg. For instance, one of the unresolved questions in relation to the AF initiation is the exact identity of the MGF (see above). The prime MGF candidate is as of yet unknown small-molecule product of the enzyme, KLUH. KLUH is a CYP78A5 expressed in the leaf base during early leaf development (Anastasiou et al., 2007; Kazama et al., 2010). Whereas overexpression of KLUH from its endogenous promoter increases organ size by delaying differentiation, *kluh* mutants are characterized by small leaves, which can be traced to fewer cells (Anastasiou et al., 2007). A detailed multiomics characterization of the *kluh* mutant recently revealed that cytokinin signaling, with its well-established function in delaying leaf senescence, was activated in KLU-overexpressing plants. Consistently, KLU-overexpressing plants exhibited significantly delayed leaf senescence and increased leaf longevity, whereas the *klu-*mutant plants showed early leaf senescence. In addition, proline biosynthesis and catabolism were enhanced following KLU overexpression owing to increased expression of genes associated with proline metabolism (Jiang et al., 2021). Despite the fact that these studies have provided considerable insight into the molecular consequences of altering KLUH expression the exact biochemical process that the protein catalyzes remains unknown. This highlights a major challenge that remains for almost all pathways of plant secondary metabolism and one that we detail in the section METABOLOMICS: TACKLING THE DARK MATTER OF THE UNKNOWN, UNKNOWNS below.

Opposite to protein centric methods, described above, are so-called small-molecule centric approaches. Such start with a metabolite bait to identify protein interactors, by default both direct and indirect, that are subsequently identified using MS. The older approaches rely on affinity resins with ligands attached to the beads (Scholten et al., 2006) or capture compounds. The latter are chemically modified ligands, which can bind covalently to their targets upon light activation and are subsequently purified using an affinity handle (Luo et al., 2009). More recent methods exploit changes in protein properties caused by small molecule binding, such as thermal stability (thermal proteome profiling [TPP]/CETSA) (Savitski et al., 2014; Franken et al., 2015), susceptibility to proteolytic cleavage (DARTS/Lip-MS) (Feng et al., 2014), or oxidation (SPROX) (West et al., 2008). Importantly, CETSA/TPP and LiP-MS can be performed using lysate and intact cells (Piazza et al., 2018), whereas LiP-MS is suitable to approximate ligand binding sites (Piazza et al., 2018). The strengths, but also limitations of the different methods have been extensively reviewed by Luzarowski and Skirycz (2019) and Venegas-Molina et al. (2021).

We propose that any of the above approaches could be used to look for protein targets of the known small molecules implicated in the regulation of leaf growth, such as for instance 12-OPDA (Omidbakhshfard et al., 2021), *N*-acetyl glutamic acid (Omidbakhshfard et al., 2021), or trehalose-6-phosphate (Fichtner and Lunn, 2021). To add to the list of candidate regulators, many of the specialized metabolites found to accumulate in the proliferating Arabidopsis leaves have recently been assigned roles outside their canonical defense function. For instance, and most notably, glucosinolates were shown to regulate both auxin transport (Katz et al., 2015) and TOR signaling (Malinovsky et al., 2017). Analogously as in the case of the ZmAN3 (see above), we speculate that the exact identity of the protein partners of the various small molecules may differ depending on the leaf growth stage. Hence, the choice of a starting material matters. Again because of their large size, we expect that maize leaves will be better suited, but some methods such as TPP and Sm-Lip require relatively little of the total protein and may also be suitable for Arabidopsis. In addition to uncovering mode of action of endogenous metabolites, small-molecule centric approaches are excellent to provide mechanistic inside into function of exogenously applied growth-promoting compounds, for example cis-cinnamic acid (Steenackers et al., 2019) or strobilurins (Van Dingenen et al., 2017), and in that provide insight into regulation of leaf growth.

Protein- and metabolite-centric approaches described above provide lists of high confidence interactors, but they are limited to the used baits. Protein centric approaches require transgenic lines, whereas metabolite centric methods by default are only suitable to known small molecules available as purified compounds. As discussed above, the latter may prove especially problematic. That is because the chemical identity of a majority of the measured small-molecules remains unresolved, and even for the compounds, we know purification or synthesis can prove highly challenging. Such limitations are circumvented by co-fractionation (CF)–MS-based methods, reviewed by Salas et al. (2020). CF–MS combines separation of protein–protein and protein–metabolite complexes with MS characterization of the obtained fractions. As such, CF–MS offers an insight into the entirety of interactomes. Separation step can entail the use of chromatography (Havugimana et al., 2012; Wan et al., 2015), native gels (Gorka et al., 2019), or centrifugation (Geladaki et al., 2019). Obtained fractions are then analyzed for their protein (Havugimana et al., 2012), metabolite (Luzarowski et al., 2021), and/or RNA composition (Smirnov et al., 2016). Obtained elution profiles are used to look for co-eluting partners, either using machine learning-based frameworks (Hu et al., 2019; McWhite et al., 2020; Zhang et al., 2020) or deconvolution and correlation (McBride et al., 2019). The main challenge of CF–MS lies in the limited separation. By default, using any separations, obtained fractions will contain more than one complex. In the past, researchers combined orthogonal chromatographic separations, such as size exclusion and ion exchange (McBride et al., 2019), and multiple CF-MS datasets (McWhite et al., 2020) to distinguish between true and coincidental interactors. A complementary strategy entails prefractions steps, for example, by enriching for certain subcellular compartments (Olinares et al., 2010), or using prefractionation columns (Hu et al., 2019).

Dissecting protein–metabolite interactomes faces substantial challenges inherent to any attempt to characterize a highly complex and dynamic biological network. Functional characterization of interactions does not keep up with the accelerating pace of identification. We envisage that in the future, this disparity will be addressed using artificial intelligence to accurately dock the ligands, genome editing technologies to target ligand binding sites, and high-throughput phenotyping approaches to assess the phenotypic consequence of the mutations. Progress in characterizing protein–metabolite interactomes is also constrained by the highly incomplete characterization of metabolomes (discussed below), even in the best studied model systems, and a limited dynamic range of proteomics. Finally, and analogously to other -omics, there is a need for better temporal and spatial resolution (discussed below).

## Metabolomics: tackling the dark matter of the unknown, unknowns

**Figure 3:**
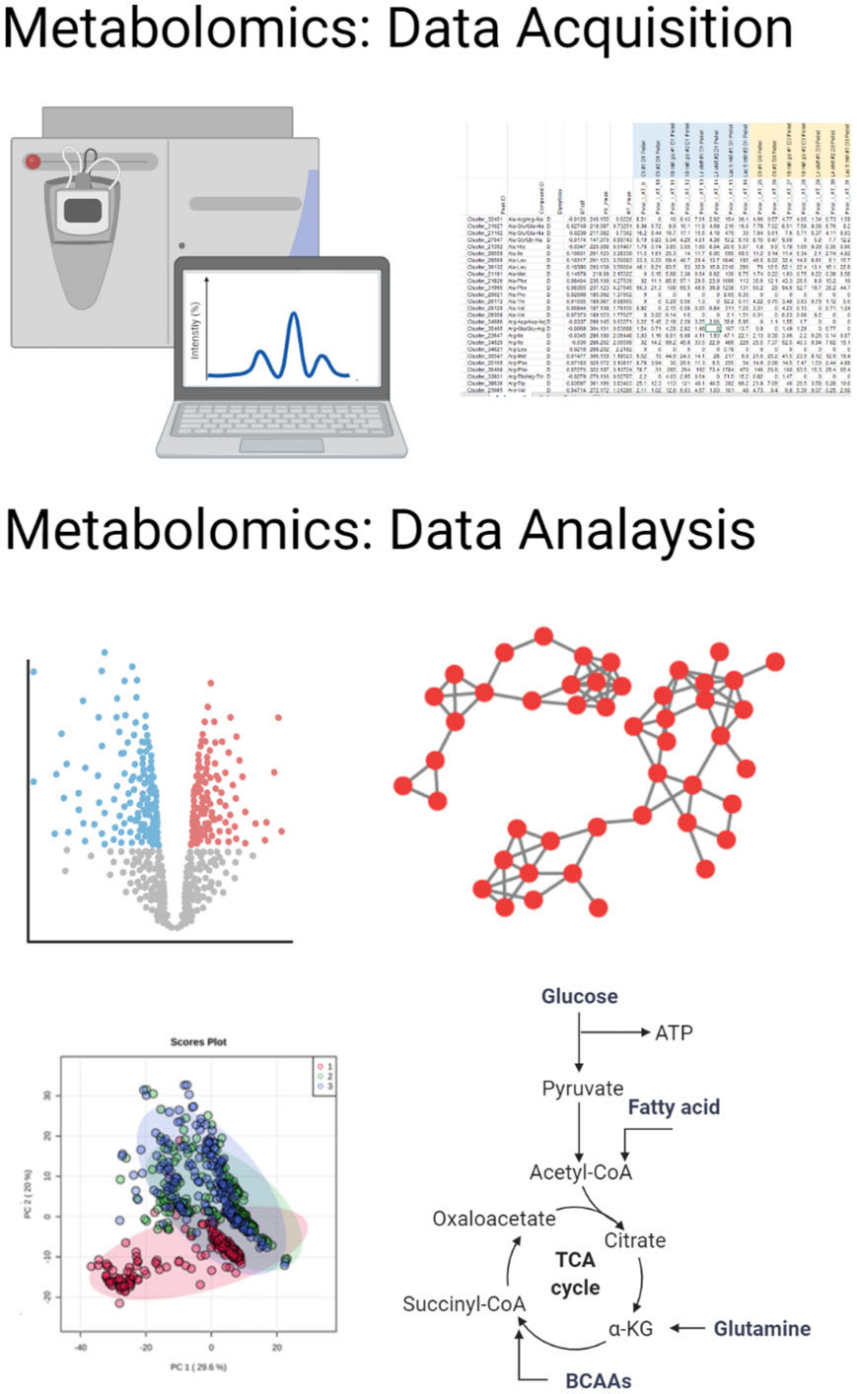
Graphical summary of MS-based metabolomics experiment. Mass spectra are processed via an appropriate software to extract information such as *m*/*z*, RT, and intensity for the measured metabolic features. Reference compounds libraries and fragmentation patterns are used to assign chemical identity to the identified features. Downstream data analysis strategies comprise descriptive statistics, network analysis, multivariate statistics and pathway analysis. Created using Biorender.com.

Although we do not know the function of the several hundred metabolites that we can measure (Alseekh and Fernie, 2018), characterizing function is not the only aspect of metabolomics that it is in urgent need of development, as expanding the coverage of metabolomics is often described as its greatest challenge (Fernie et al., 2004; Saito and Matsuda, 2010a, 2010b). Before detailing possible solutions to this, it is perhaps prudent to first describe the state-of-the-art. Current estimates for the number of metabolites extant in the plant kingdom are of the order of 200,000 to 1 million (Fang et al., 2019; Wang et al., 2019), with any singles species thought to contain between thousands and tens of thousands of metabolites (Fernie et al., 2004). Presently, the most widely utilized platforms for metabolomics analysis are GC–MS and LC–MS which are able to identify and quantify up to a couple of hundred primary metabolites, a similar number of lipids and a couple of thousand secondary metabolites (Alseekh and Fernie, 2018), and Figure 3 compares the different MS based metabolomics data analysis strategies. By contrast, the platforms record in excess of 400,000 prominent molecular features respectively suggesting that in theory we could identify far more metabolites than we currently do (Alseekh et al., 2021). Given that the promise offered by LC–MS coupled to NMR is yet to materialize (Fernie et al., 2004), two distinctive experimental approaches are being taken currently in order to enhance coverage, namely the running of large quantities of purified standards (Tsugawa et al., 2015; Shahaf et al., 2016) and following the metabolic fate of heavy isotope labels (Giavalisco et al., 2009; Nakabayashi and Saito, 2020). The former approach is arguably best demonstrated in plants with the WEIZMASS library constituting information on some 3,500 compounds (Shahaf et al., 2016). The results are even more striking with MS-DIAL, which, using an enriched LipidBlast library, identified a total of 1,023 lipid compounds in algae (Tsugawa et al., 2015). However, as yet this approach while offering considerable additional information has only made minor inroads into the dark matter of polar metabolomics. The same is true of the second approach which involves incubating tissue in heavy labeled compounds and tracking the mobilization of the stable isotope through metabolism. This approach has the advantage of providing clear indication as to whether a molecular feature is a metabolite (as opposed to a contaminant) and gives extra accuracy for high mass resolution measurements, but as mentioned above for the external standards has as yet failed to account for the majority of unknown metabolites.

In addition to these experimental approaches, a multitude of computational advances offer much promise (Aron et al., 2020; Jarmusch et al., 2020; Nothias et al., 2020; Aksenov et al., 2021; Dührkop et al., 2021; Schorn et al., 2021; Tripathi et al., 2021). These examples only cover a tiny proportion of what is available but, in our opinion, provide excellent examples of what is currently, and potentially achievable. The articles we highlight are all authored or co-authored by Pieter Dorrestein who in community driven efforts is pioneering many of these approaches. Many of these approaches are based on molecular networking which aligns experimental spectra against one another connecting related molecules by their spectral similarity (Nothias et al., 2020). This is a powerful approach that has largely been used for analysis of LC–MS data of nonplant systems, however, recently a version for use with GC–MS has been developed (Aksenov et al., 2021) and it is beginning to be adopted by plant scientists (Morreel et al., 2014; Naake and Fernie, 2019; Perez de Souza et al., 2019). The second approach is highly similar conceptually but relies purely on data from MS/MS fragmentation patterns. The most recent updates to such approaches include the software tools CANOPUS (Dührkop et al., 2021) and Qemistree (Tripathi et al., 2021) which utilize deep neural networks to predict compound classes from spectral data and use ecological tools of tree-guided data exploration to compare metabolomics datasets, respectively. Finally, in the context of this article, the recent publication of a community resource for paired genomic and metabolomic data mining is highly pertinent (Schorn et al., 2021). Particularly so, in light of the demonstration that proteomic data from Arabidopsis leaf development could be paired to transcriptomics data from an entirely different experiment (Omidbakhshfard et al., 2021). As we have previously argued in a metabolomics white paper (Alseekh et al., 2021), FAIR compliant reporting of metabolomics data would aid massive value to the datasets since it would allow them to be compared and contrasted with data obtained using different analytical platforms thereby facilitating deeper understanding of the shifting molecular (Van Leene et al., 2010) networks accompanying developmental transitions.

## Toward better temporally and spatially resolved omics

Whilst the above studies undoubtably provided a wide range of insights into the co-ordination of metabolism with growth across the developmental process, it is important not to over-interpret the results from these studies since, as has previously been discussed, there are several reasons why there is little apparent overlap between the various omics layers (Fernie and Stitt, 2012; Walley et al., 2016). In a direct comparison that is pertinent to the biological examples given above, Walley et al. found that there was relatively little overlap between transcriptional and proteomic networks in a developmental atlas of maize but that the use of both techniques was particularly useful as a strategy to increase confidence in the obtained gene regulatory networks. There are, however, many reasons for a lack of concordance between the omics levels with some of these being related to the stability of the individual entities and their ease of extraction and others being related to time lags between the perception of development/environmental cues and how rapidly the response to these is propagated through the various levels of cellular information. Given that the theory underlying this has been discussed in detail elsewhere (Fernie and Stitt, 2012), we will not reiterate it here suffice to say deeper understanding of the networks governing leaf development will most certainly require higher resolution datasets particularly in the case where mechanistic information concerning the causality of changes is required. To gain a more comprehensive understanding of the metabolic shifts occurring on leaf development in both C3 and C4 leaves, we arguably need higher resolution at the temporal and spatial level. The need for the former is easily met via denser sampling, however, as described above reasonable datasets have already been gathered at this level (Pick et al., 2011; Wang et al., 2014; Czedik-Eysenberg et al., 2016; Omidbakhshfard et al., 2021). The need for higher spatial resolution is thus more acute. A great example is the recently published single cell-transcriptomics map representing 14 different cell populations from young Arabidopsis leaves harvested from seedlings grown under control and mild-drought conditions (Tenorio Berrío et al., 2021). The presented dataset containing proliferating and differentiating cells was used to identify markers for the different cell types, revealed distinct expression patterns for indole and aliphatic glucosinolate biosynthetic enzymes, and demonstrated tissue-specific responses to drought. That said, while not as developed as single cell (epi)genomics techniques (Luo et al., 2020), several attempts have been made in this direction that warrant discussion. Among these are the use of laser capture microdissection (Schad et al., 2005), isotope labeling (Szecowka et al., 2013; Rossi et al., 2017), and fluorescence-activated cell sorting (Moussaieff et al., 2013), which have been used to gain information concerning cell type specific metabolomes whilst both silicon oil centrifugation and nonaqueous fractionation have been used to gather information concerning the sub-cellular levels of metabolites (Tohge et al., 2011; Szecowka et al., 2013; de Souza et al., 2020). In this section, we will detail what has or potentially could be learnt on the application of these techniques to the study of leaf development before highlighting state-of-the-art image based methodologies that provide exquisite temporal resolution of metabolism (Dong et al., 2020; Pareek et al., 2020; Zhang and Fernie, 2020).

Laser-capture microdissection allows careful control of which cells are harvested. It works by ablating the area around the cells in question using a laser and catapulting the remaining tissue into a plastic tube for collection. Although still mainly used for transcriptomics (Luo et al., 2019; Martinez et al., 2021; Thiel et al., 2021), this approach has been adapted for proteomics (Liang et al., 2018; Abreu et al., 2020) and metabolomics (Schad et al., 2005; Gaude et al., 2015; Huang et al., 2016). As well as the early demonstration of this approach in defining the metabolites of Arabidopsis phloem (Schad et al., 2005), it has more recently been used to characterize *Cayrartoa japonica* (“bushkiller”) leaves and AM symbiosis in *Medicago truncatula* (“barrelclover”) (Gaude et al., 2015; Huang et al., 2016). While not really used yet for increasing understanding of leaf development it clearly could have utility in this, especially in tissues with large leaves. While these approaches are direct and highly sensitive, they suffer from the drawback that they are incredibly laborious. Partially driven by this fact, indirect but more facile methods to gain spatial information have been developed based on heavy isotope labeling. These range from very indirect methods that simply use labeling kinetics of various metabolite pools following isotope tracer experiments to infer the presence of metabolically active and inactive pools (Szecowka et al., 2013;Xu et al., 2021) to the use of isotope labeling in cell type specific marker proteins such as GFP in order to provide insight into the metabolism of cells in which these markers are expressed (Rossi et al., 2017). A final approach that has been taken to address cell type specificity latched on to the fluorescence-activated cell sorting approach that has been much used in the characterization of the Arabidopsis root transcriptome (Brady et al., 2007). In doing so (Moussaieff et al., 2013) were able to identify cell-specific accumulation of a range of glucosinolates, phenylpropanoids, and dipeptides. However, it should be noted that given that the FACS approach is quite lengthy this approach is only valid for highly stable metabolites such as those profiled in their study. The other approach to gain cell specific information has been to target easy-to-isolate cell types—two for which a particularly large battery of studies exist are glandular trichromes (Fan et al., 2019; Leong et al., 2019; Galdon-Armero et al., 2020; Leong et al., 2020) and stomatal guard cells (Daloso et al., 2016; Medeiros et al., 2018; David et al., 2020; Zhu et al., 2020)—although in the latter case information is often on guard-cell enriched fractions rather than pure populations of guard cells.

Whilst diverse methods are available for cell-specific study those for enhancing subcellular aspects of metabolism are almost exclusively reliant on the differential centrifugation of the subcellular compartments. Two major approaches exist—silicon oil centrifugation of protoplasts (Wirtz et al., 1980) and the nonaqueous fractionation technique (Stitt et al., 1985). Both methods were developed in animal research and widely adopted for plants in the 1980s (Wirtz et al., 1980; Lilley et al., 1982; Stitt et al., 1985; Gerhardt et al., 1987). While silicon oil centrifugation has been used in a few metabolomics studies—arguably most successfully in delineating the *Hordeum vulgare* (barley) vacuolar storage metabolome (Tohge et al., 2011), nonaqueous fractionation which utilizes material in its native form rather than in the artificial protoplast state has received far greater utility (Krueger et al., 2011). Indeed, recent application of this approach has led to considerable advances in our understanding of the regulatory control of *Solanum tuberosum* (potato) tuber starch biosynthesis (Tiessen et al., 2002), as well as nonorganellar sub-compartments (Krueger et al., 2011), in addition to facilitating much better modeling of metabolic flux (Szecowka et al., 2013; Arrivault et al., 2017). Furthermore, it could be demonstrated that metabolites sequester within nonmembrane organelles. Stress granules (SGs) are aggregates of proteins and RNAs, which form in response in stress, and in plants SGs can be found in both the cytosol and plastid. Characterization of the cytosolic and plastidial SGs led to the identification of tens of proteins but also of the metabolites that sequester within SGs, most likely via PMIs (Kosmacz et al., 2019; Kosmacz and Skirycz, 2020). Identified metabolites include amino acids such as proline and glutamate, nucleotides and phospholipids. The role of metabolite sequestration within SGs is as of yet unknown.

## Conclusions and future perspectives

The systems biology of leaf growth is relatively mature, particularly in Arabidopsis and maize. Indeed, several key regulators have been identified and validated and a plethora of further candidate targets have been proposed. Recently, the adoption of multiomics approaches and in particular inclusion of metabolomics has shed further light on this process. However, as illustrated by the example of KLUH above, whilst current technologies illuminate some of the molecular responses to deficiencies providing important hints toward their function they do not always lead to exact mechanistic understanding at the reaction level. That said multiple recent developments in metabolomics as well as recently developed techniques that provide insight into metabolite–protein interactions hold great promise for the extension of such catalogs as well as providing insight into the functional mechanism which underly them. Despite its limitations, to our knowledge, CF–MS provides a unique strategy for a proteome- and metabolome-wide characterization of the interactomes. We propose that building PPI and PMI maps across the different growth stages, especially the transition zone, will prove immensely useful in providing insight into the regulation of leaf growth, such as to identify additional small-molecule regulators of the major transitions, from proliferation to expansion and from expansion to maturity. Identifying such regulation, as well as better defining those described above represents an exciting opportunity to improve our understanding as to how small-molecule protein interactions impact leaf growth. The importance of such findings should not be underestimated since as opposed to the situation in for example yeast or humans very few metabolite receptors have been identified in plants to date. This fact notwithstanding as we discussed above we have now accrued a massive amount of information concerning the molecular shifts that occur during leaf growth in both C3 and C4 species with mechanistic understanding being defined for several of these. Thus, despite the fact that, as detailed in the Outstanding Questions box, several pertinent questions remain to be addressed, we feel that the application of recently developed technologies alongside those currently in development will allow us to rapidly fill these gaps.

*Conflict of interest statement*. Authors declare no conflict of interest. 

## Notes

A.S. and A.R.F jointly wrote the paper and prepared figures.

The author responsible for distribution of materials integral to the findings presented in this article in accordance with the policy described in the Instructions for Authors (https://academic.oup.com/plphys/pages/general-instructions) is: Aleksandra Skirycz (as4258@cornell.edu).
